# Protective barrier properties of Rhinosectan^®^ spray (containing xyloglucan) on an organotypic 3D airway tissue model (MucilAir): results of an in vitro study

**DOI:** 10.1186/s13223-017-0209-6

**Published:** 2017-08-10

**Authors:** Barbara De Servi, Francesco Ranzini, Núria Piqué

**Affiliations:** 1 VitroScreen Srl, Via Mosè Bianchi 103, 20149 Milan, Italy; 2 Department of Microbiology and Parasitology, Diagonal Sud, Facultat de Farmàcia, Universitat de Barcelona (UB), Edifici A, Av Joan XXIII, 08028 Barcelona, Spain

**Keywords:** Nasal obstruction, Rhinitis, Rhinosinusitis, Orgatypic 3D airway tissue model (MucilAir), Rhinosectan^®^, Xyloglucan, Barrier properties, Allergy, Preventive measures

## Abstract

**Background:**

To evaluate barrier protective properties of Rhinosectan^®^ spray, a medical device containing xyloglucan, on nasal epithelial cells (MucilAir).

**Methods:**

MucilAir-Nasal, a three-dimensional organotypic (with different cell types) airway tissue model, was treated with the medical device Rhinosectan^®^ (30 µL) or with controls (Rhinocort—budesonide—or saline solution). The protective barrier effects of Rhinosectan^®^ were evaluated by: TEER (trans-epithelial electrical resistance) (preservation of tight junctions), Lucifer Yellow assay (preservation of paracellular flux) and confocal immunofluorescence microscopy (localization of tight junction proteins).

**Results:**

Exposure of MucilAir with Rhinosectan^®^ protected cell tight junctions (increases in TEER of 13.1% vs −6.3% with saline solution after 1 h of exposure), and preserved the paracellular flux, even after exposure with pro-inflammatory compounds (TNF-α and LPS from *Pseudomonas aeruginosa* 10). Results of confocal immunofluorescence microscopy demonstrated that, after treatment with the pro-inflammatory mixture, Rhinosectan^®^ produced a slight relocation of zona occludens-1 in the cytosol compartment (while Rhinocort induced expression of zona-occludens-1), maintaining the localization of occludin (similarly to negative control).

**Conclusions:**

Results of our study indicates that Rhinosectan^®^ creates a protective physical barrier on nasal epithelial cells in vitro, allowing the avoidance of allergens and triggering factors, thus confirming the utility of this medical device in the management of nasal respiratory diseases, as rhinitis or rhinosinusitis.

## Background

Nasal obstruction is one of the most common reasons by which patients visit their primary care providers [1]. Often described by patients as nasal congestion or the inability to adequately breathe out of one or both nostrils during the day and/or night, nasal obstruction commonly interferes with a patient’s ability to eat, sleep, and function, thereby significantly impacting quality of life [1]. The most common causes of nasal obstruction are rhinitis (allergic and non-allergic), rhinosinusitis, drug-induced nasal obstruction and mechanical/structural abnormalities [1].

Currently, with the advent of high levels of antibiotic resistance [2, 3], different medical societies have issued recommendations to use non-pharmacological measures whenever possible for acute respiratory tract infections [4, 5]. Moreover, there is currently an increasing interest to avoid secondary effects with chronic pharmacological measures; reducing the use of intranasal glucocorticosteroids and antihistamines [6, 7].

In the case of rhinosinusitis, based on a narrative literature review, The American College of Physicians (ACP) and the Centers for Disease Control and Prevention (CDC) recently published advice for high-value care on the appropriate use of antibiotics for acute respiratory tract infections, concluding that clinicians should reserve antibiotic treatment for acute rhinosinusitis in patients with persistent symptoms for more than 10 days, high fever and purulent nasal discharge or facial pain lasting for at least 3 consecutive days, or worsening symptoms after a typical viral illness that lasted 5 days and had initially improved (“double-sickening”) [5].

In the case of rhinitis, avoidance of allergens and other triggering factors should be indicated when possible and should be an integral part of the management strategy, according to the clinical and practical recommendations of ARIA (allergic rhinitis and its impact on asthma) guidelines [7, 8].

In this context, a non-pharmacological approach is to employ measures that create a mechanical barrier over the sinonasal mucosa, with the aim of reducing contact between allergens, irritants, pathogens and triggering factors and the mucosa [9, 10].

The utility of these measures is also supported by recent data suggesting that the epithelium with its tight junctions is considered tight under normal conditions but it can be abnormally permeable, with a decreased presence of tight junctions, in pathogenic conditions (the “hyperpermeability hypothesis”), as allergic rhinitis [9, 11–13] or rhinosinusitis [14, 15], or also in contact with high levels of contamination (such as traffic-related air pollutants) [16]. Since epithelial barrier defects are linked with chronicity and severity of airway inflammation, restoring the barrier integrity may become a useful approach in the treatment of allergic diseases [13].

In this regard, non-pharmacological barrier measures, such as nasally applied cellulose powder (which hygroscopically takes up water to form a gel on the mucosa) [17, 18] and lipid microemulsions [7, 19] have been shown to reduce the symptoms of allergic rhinitis in double-blind, placebo-controlled studies, while an observational study has provided preliminary evidence of efficacy for a liposomal nasal spray [10, 20].

Rhinosectan^®^ spray (Novintethical Pharma, SA; Pambio-Noranco, Lugano, Switzerland) is a medical device, containing xyloglucan as main ingredient, developed to restore the physiological functions of the nasal epithelial mucosa forming a film that protects the mucosa from different pathogens and allergens. Rhinosectan^®^ is specifically formulated for the control and reduction of the symptoms related to rhinorrea and sinus congestion due to different aetiologies, such as rhinitis (seasonal, perennial allergic, infectious or vasomotor rhinitis), sinus congestion due to cold or flu; and as symptomatic relief associated to the treatment of nasal polyps and acute sinusitis. Xyloglucan is a natural hemicellulose extracted from the seeds of the tamarind tree (*Tamarindus indica*), which forms a bio-protective film. In in vitro and in vivo studies, we have demonstrated the barrier properties of xyloglucan, avoiding the contact with pathogenic bacteria and bacterial products, being currently used as alternative non-pharmacological alternatives in intestinal and urinary tract alterations [21–25].

Moreover, in a recent randomized, double-blind study in 40 patients with rhinosinusitis, itching, nasal congestion or continuous sneezing, the administration of the xyloglucan-based spray during 2 weeks reduced rhinorrhoea, itching, TNSS (total nasal symptom score) and the severity of rhinosinusitis significantly compared with a physiological saline nasal spray [10].

Based on these favourable results on the nasal mucosa, we have designed the present in vitro study to assess the barrier properties of Rhinosectan^®^ spray in the airway tissue model MucilAir. Results obtained confirm that Rhinosectan^®^ spray is also able to create a protective barrier on nasal cells, which avoids the contact of mucosal cells with triggering factors.

## Methods

### Products

Rhinosectan^®^ spray contains physiological saline solution, mehylsulfonylmethane and xyloglucan, extracted from the seeds of the tamarind tree (*Tamarindus indica*), The product was kindly provided by Novintethical Pharma SA (Switzerland).

The protective effect of Rhinosectan^®^ spray was compared with the already marketed medicinal product Rhinocort^®^ nasal spray, containing the glucocorticoid budesonide as drug substance.

As negative control, saline solution (0.9% NaCl) (Eurospital, Trieste, Italy) was used.

### Cells and reagents

MucilAir-Nasal (Epithelix Sàrl, Geneva, Switzerland) [26] is an organotypic 3D airway tissue model in which nasal epithelial cells are cultured at the air–liquid interface (ALI) and capable of differentiating to form a pseudostratified cell layer containing mucus-secreting goblet cells and ciliated columnar cells [26, 27]. The ALI allows a direct administration of an aerosol onto the apical surface, a situation resembling aerosols exposure of the in vivo respiratory system [26]. Moreover, the epithelium is nourished by a culture medium from the basolateral surface [26, 27].

MucilAir-Nasal, a morphologically and functionally differentiated nasal epithelium, was used as nasal mucosa model. The epithelia are cultivated on microporus filters at air–liquid interface and they are fully differentiated. They can be maintained at a homeostatic state for more than one year. Typical ultra-structures of the human airway epithelium are observed: tight junctions, cilia, basal cells and mucous cells.

In the present study, batch MP0006 was used. Immediately after arrival in the laboratory, the Mucilair tissues were rapidly transferred to a 24-well plate previously filled with 0.7 mL of the specific MucilAir maintenance medium (Epithelix) at room temperature. The wells were placed in an incubator at 37 °C, 5% CO_2_ and saturated humidity overnight.

Other reagents used were Lucifer Yellow (LY) (Sigma-Aldrich, Steinheim, Germany).

### Evaluation of preservation of tight junctions of nasal epithelial cells (TEER resistance)

The effects of Rhinosectan^®^ spray in preserving the tight junctions of nasal epithelial cells were evaluated in MucilAir cells using Trans-Epithelial Electrical Resistance (TEER). TEER is the measure of movement of ions across the paracellular pathway regulated by polarized plasma membranes surfaces and by cell-to-cell tight junctions, which together prevent movement of solutes and ions across the epithelia. TEER is an indirect assessment of tight junction stability and, consequently, is a direct measure o the barrier linked both to the structure and to epithelial thickness.

Cell monolayers were treated with 30 µL of each product (Rhinosectan^®^ spray, Rhinocort and the negative control saline solution), in triplicates, in a 24 well plate containing 0.7 mL of saline solution/well during 15 min or 1 h. Both untreated cell-monolayers and transwells with the filter insert without cells were used as controls.

TEER was applied to measure the barrier integrity by placing the appropriate electrodes in the apical (AP) and basolateral (BL) positions according to the manual instructions (Millicell^®^ ERS meter, Millipore, Bedford, MA, USA). TEER measurements were carried out the day before the addition of Rhinosectan^®^ (basal value, t0) and after 15 min and 1 h of exposure (removed by suction) and after an additional hour of recovery. Final TEER values (Ω × cm^2^) of cell-monolayers were obtained after subtracting the TEER value produced by the filter insert without cells.

### Evaluation of barrier properties (permeability) by Lucifer Yellow assay

The effects of Rhinosectan^®^ in preserving the paracellular flux within the mucosal barrier model were evaluated in MucilAir cells by LY assay, which was performed before and after treatment to measure the degree of porosity of intercellular tight junctions of epithelial cells.

Briefly, cell monolayers were pre-treated with 30 µL of the products (Rhinosectan^®^, Rhinocort or saline solution), in duplicates. Untreated cells were used as controls. The products were added on the apical part of the inserts and maintained during 2 h; then, cell monolayers were stimulated with a mix of pro-inflammatory compounds (TNF-α 500 ng/mL plus LPS from *Pseudomonas aeruginosa* 10, 0.2 mg/mL) and incubated overnight (16 h). After the overnight exposure, the medium was replaced with fresh neutral medium and the culture was incubated during one hour (recovery period).

After exposure of the product, 0.2 mL/well of LY (100 µM dissolved in HBSS—Hanks’ Balanced Salt Solution-buffer) were applied in the apical part compartment of the cell monolayer, and 0.5 mL of HBSS was applied in the basolateral compartment. Cells were then incubated for 1 h at 37 °C, 95% humidity and 5% CO_2_.

After incubation, the paracellular flux of LY from the apical part to the basolateral compartment was measured by fluorescence (relative fluorescence units, RFU) using spectrofluorimeter (Tecan Infinite M200) at 428 nm excitation and 535 nm emission. LY flux was calculated with the following formula:

$$LY\;Flux = (RFU_{BL} /RFU_{AP} ) \times 100,$$

where RFU_BL_ are fluorescent units detected at the basolateral compartment and RFU_AP_ are fluorescent units detected at the apical part compartment.

### Localization of tight junction proteins upon exposure of Rhinosectan^®^ (confocal immunofluorescence microscopy)

Confocal immunofluorescence microscopy allows the visualization of a specific protein or antigen in cells or tissue, in which a secondary antibody labeled with fluorochrome is used to recognize a primary antibody.

Immunofluorescence stained samples were examined under a fluorescence microscope or confocal microscope (Leica TCS SPE confocal laser scanning, Leica, Germany).

The specificity of the immuno-localisations was demonstrated in the slides where the primary and secondary antibodies were replaced with saline solution.

The studied products (Rhinosectan^®^, Rhinocort or saline solution) were added on the apical part of the inserts and maintained during 2 h; then, cell monolayers were stimulated with an inflammatory mix (TNF-α 500 ng/mL plus LPS from *Pseudomonas aeruginosa* 10, 0.2 mg/mL) and incubated overnight (16 h). After the overnight exposure, the medium was replaced with fresh neutral medium and the culture was incubated during 1 h (recovery period).

To assess localization of occludin, the mouse monoclonal antibody anti-occludin (OCLN antibody 33-1500; Invitrogen Antibodies, California, USA), diluted at 2 µg/mL, was added and incubated for 1 h at room temperature. The secondary antibody Alexa Flour 488 goat anti-mouse (A10-680, Invitrogen Antibodies) was then added and the nuclei was stained with Hoeschst (Sigma-Aldrich).

To assess localization of zona occludens, the rabbit polyclonal antibody (Invitrogen, 61-7300), diluted at 2 µg/mL, was added and incubated for 1 h at room temperature. The secondary antibody Alexa flour 555 donkey anti-rabbit (Invitrogen, A31572) was then added and the nuclei was stained with Hoeschst.

After exposure of the studied products (during 15 min and one hour plus one hour of recovery and after an overnight exposure), MucilAir tissues were fixed with ethanol (30 min) at 4 °C and then with acetone (3 min) at room temperature. Cells were incubated with 1% BSA for 30 min to block unspecific binding of the antibodies.

Primary occludin mouse monoclonal antibody and zonulin-1 polyclonal rabbit antibody were applied for 1 h, followed by incubation with the corresponding secondary antibody. The slices were stained with Hoechst for nuclei staining and they have been mounted with glycerol 90% in PBS and sealed with nail varnish.

The slides were examined under Leica TCS SPE confocal laser scanning microscope (Leica Microsystems, Wetzlar, Germany) using a sequential scan procedure during image acquisition of double label sections. Confocal images were taken through the z axis of the sections. Images from individual optical planes and image projections of stacks of serial optical planes were analyzed by confocal software (Multicolor Package, Leica).

### Statistical analysis

A descriptive analysis of quantitative data was performed. Mean and standard deviation of TEER and LY (%) values were calculated from Rhinosectan^®^-, Rhinocort- and saline-treated and untreated cell monolayers.

The Student’s T test was used to compare results between two conditions. p values lower than 0.05 were considered significant.

## Results

### Rhinosectan^®^ contributes to preserve tight junctions of nasal epithelial cells (TEER evaluation)

Upon exposure with Rhinosectan^®^ during one hour and one hour plus recovery (1 + 1 h), TEER values increased by a 13.1 and 8.0%, respectively, while with saline solution TEER values decreased by −6.3 and −3.4%, respectively, with statistically significant differences between Rhinosectan^®^ and saline solution (p < 0.01 after 1 h and p < 0.05 after 1 + 1 h). Upon an acute exposure of 15 min, the reduction in TEER values was more notable in the control samples than in the Rhinosectan^®^ treated samples (−35.9 vs −16.6%) (Table 1).

**Table 1:** Normalized TEER values (% TEER), considering the basal values equal to 100% (T = 0)

Product % increase/decrease of TEER values	Time			
	15 min	15 min + 1 h	1 h	1 + 1 h
Saline solution	−35.9	−20.1	−6.3	−3.4
Rhinosectan^®^	−16.6 ± 26.3	−24.6 ± 36.1	13.1 ± 4.8	8.0 ± 11.7
p value Saline solution vs Rhinosectan^®^	>0.05	>0.05	<0.01	<0.05

TEER values were obtained after 15 min + 1 h and after 1 + 1 h (in duplicates for Rhinosectan^®^ and in simplicates for the negative control (saline solution)

### Protective properties of Rhinosectan^®^ to preserve the paracellular flux

**Fig. 1:**
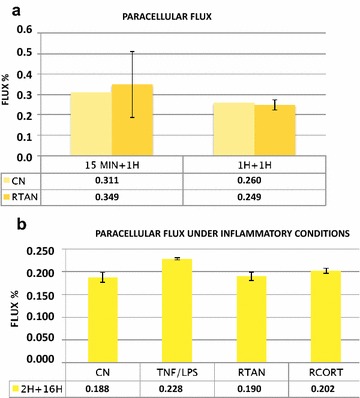
Preservation of paracellular flux by Rhinosectan^®^ between the apical and basolateral compartments of MucilAir. **a** LY permeability after 15 min and 1 h (plus 1 h of recovery) (LY flux (%) values). **b** LY permeability after 2 h of pre-treatment and 16 h of exposure to pro-inflammatory compounds (LY flux (%) values)

Rhinosectan^®^ did not alter cell permeability of MucilAir cells, maintaining the paracellular flux between AP and BL compartments of treated cells after 15 min and 1 h of exposure, with LY fluxes similar to the saline solution (0.249% with Rhinosectan^®^ vs 0.260% with saline solution after 1 + 1 h) (Fig. 1a), and also similar to untreated cells, thus reflecting the integrity of the mucosal barrier.

In the presence of pro-inflammatory compounds (TNF-α and LPS), cell permeability in the negative control (saline solution) increased (from 0.188% in absence to 0.228% in the presence of pro-inflammatory compounds), while cells treated with Rhinosectan^®^ maintained low permeability levels (0.188%). The exposure with budesonide (Rhinocort) produced LY fluxes higher than Rhinosectan^®^ (0.202%, p < 0.05) (Fig. 1b).

### Re-location of zonula occludens-1 protein in the cytosol upon exposure to Rhinosectan^®^

**Fig. 2:**
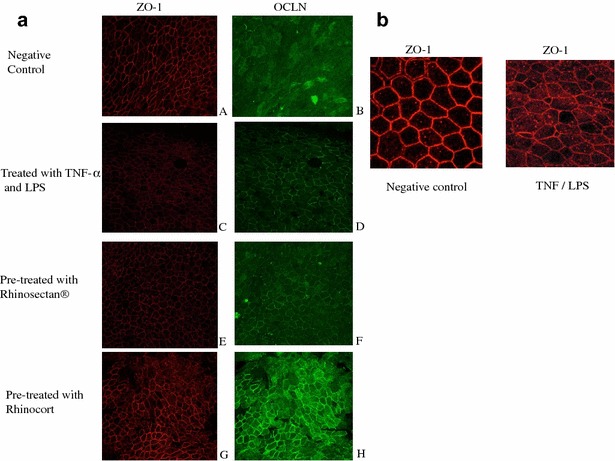
Evaluation by confocal immunofluorescence microscopy of localization of zonula occludens-1 and occludin in MucilAir cells. **a** Observation after 2 h of pre-treatment and 16 h of exposure to pro-inflammatory compounds. *ZO-1* zona occludens-1, *OCL* occludin. **b** Evaluation by confocal immunofluorescence microscopy of localization of zonula occludens-1 in MucilAir cells, where the intensity of the staining of the inflammed tissue was increased to better visualize the different localization of the proteins. *ZO-1* zona occludens-1

Results of confocal immunofluorescence microscopy showed that, in the negative control samples, zonula occudens-1 was homogeneously distributed in the membrane, while occludin was homogeneously distributed in both membrane and the cytoplasmatic compartment of MucilAir cells (Fig. 2).

After exposure during 16 h with pro-inflammatory compounds, a decrease in the expression of zonula occludens-1 in the membrane and occludin, which was predominantly expressed at membrane level with a functional role of reaction to the inflammatory status, was observed (Fig. 2). Many intracellular vesicular pools of zonula occludens-1 were present compared to negative control (Fig. 2b).

Rhinocort pretreatment induced zonula occludens-1-protein expression recovery in the tight junction structure, with higher intensity compared to negative control (Fig. 2). Occludin protein also increased in the membrane compartment with higher intensity (Fig. 2).

Rhinosectan^®^ pre-treatment induced a relatively slight increase of either zonula occludens-1 and occludin proteins expression in the membrane compartment, compared to the inflammed tissue.

Occludin localization appeared modified compared to positive control (higher intensity) and was more similar to negative control localization (both cytoplasmatic and membrane localization) (Fig. 2).

## Discussion

Currently, the role of the mucosal barrier integrity is gaining increasing interest among scientists, as a primary prevention of different diseases, as intestinal or respiratory disorders [9, 28].

The mucosal barrier of the upper respiratory tract, including the nasal cavity, which is the first site of exposure to inhaled antigens, plays an important role in host defense in terms of innate immunity and is regulated in large part by tight junctions of epithelial cells. Tight junction molecules are expressed in both M cells and dendritic cells as well as epithelial cells of upper airway [14].

In the present study, we have demonstrated that the exposure with Rhinosectan^®^, mainly due to xyloglucan, contributed to the preservation of tight junctions, as demonstrated by an increase of TEER values across time.

Maintenance of stability and electrical resistance of an epithelium is critical for essential physiological processes, therefore significant changes in TEER may represent an early expression of cell damage and it can be considered a complementary parameter.

In fact, a decreased TEER has been found in biopsy specimens from patients with chronic rhinosinusitis with nasal polyps along with an irregular, patchy, and decreased expression of the tight junctions molecules occludin and zonula occludens 1 [15], thus suggesting the beneficial role of products of Rhinosectan^®^ with the capacity of increase TEER values.

The protective effects of xyloglucan have also been observed in models of intestinal mucosa. On CacoGoblet cell monolayers, exposure with xyloglucan (in the medical device Utipro) produced higher TEER values in comparison with untreated cells [21].

In concordance with TEER results, we have also confirmed that the exposure to Rhinosectan^®^ did not alter the paracellular flux, even after treatment with pro-inflammatory compounds (TNF-α and LPS). These results indicate that the presence of bio-protective film produced by xyloglucan avoids the contact of these triggering factors with the nasal mucosal layer.

Previously, the nasal epithelium was considered only as a barrier, but now it is considered as a central player in controlling the immune function release of innate cytokines-promoting Th2 responses and the activation of local dendritic cells [29–31]. The exposure of airways to aeroallergens induces a rapid release of cytokines from the epithelial cells into the airway lumen and initiates an allergic immune response [11, 32–34].

The presence of xyloglucan, therefore, is thought to avoid the contact of epithelial cells with aeroallergens, as pollen, and also with the released cytokines (as TNF-α), thus attenuating the allergic response. Moreover, the avoidance of contact of bacterial LPS with the monolayers is thought to attenuate the LPS-induced inflammation of nasal epithelial cells, and, therefore, the inflammatory process mediated by the bacterial LPS from Gram negative bacteria [34]. In previous studies, we have also demonstrated that xyloglucan is able to prevent LPS-mediated alteration of tight junction permeability in a model of Caco-2 cells [21].

Results obtained by confocal immunofluorescence microscopy also highlight the barrier properties of Rhinosectan^®^, with low affectation of the tight junction proteins zonula occludens-1 and occludin. This non-pharmacological effect of Rhinosectan^®^ contrasts with the known pharmacological effect of the active substance of Rhinocort (the glucocorticoid budesonide), which counteracted the pro-inflammatory effects of LPS and TNF-α, increasing the expression of both tight junctions proteins, as already described for glucocorticoids [12, 35, 36]. These results are expected according to the pharmacological activity of budesonide. The tight junctions proteins appeared reinforced either at structure level and for their functional role in repairing the induced damage compared to negative control.

The product Rhinosectan^®^, which after 1 h of treatment promoted a slight TEER increase compared to negative control (slight film forming activity), confirmed its action on the stabilization of occludin (at both cytosolic and membrane level), which has a crucial role as a functional component of the tight junction, maintaining the intramembrane diffusion barrier and enhancing the functional (adaptive role) of the tight junctions structure in assuring the barrier function.

The barrier properties of Rhinosectan^®^, maintaining the tight junction structure, is of relevance, taking into account that tight junction defects have recently been associated with asthma and chronic rhinosinusitis, although poorly understood in allergic rhinitis [12].

We should note that our results are in concordance with the favourable results obtained in the clinical trial in patients with rhinosinusitis [10], thus highlighting the validity of the in vitro MucilAir model. Tissue cultures and 3D tissue models are recognised as being a sensitive and reliable model for in vitro toxicology and pharmaco-toxicological testing in order to replace animals (thus minimizing their use). They have been considered suitable alternatives for safety and efficacy assessment of active substances and medicinal products [37].

We consider this model will be also of usefulness for further studies with Rhinosectan^®^, for example, to assess its barrier properties to avoid the contact with different aero-allergens as pollen or dust mites, as a model of allergic rhinitis.

## Conclusions

In conclusion, in the present study, we have demonstrated that Rhinosectan^®^ creates a protective physical barrier on nasal epithelial cells in vitro, which avoids the contact of mucosal cells with pro-inflammatory compounds. Therefore, these results confirm the utility of Rhinosectan^®^ in the management of nasal respiratory diseases.

## Abbreviations

3D: 3-dimensions
ACP: The American College of Physicians
ALI: air–liquid interface
AP: apical
ARIA: allergic rhinitis and its impact on asthma
BL: basolateral
CDC: Centers for Disease Control and Prevention
HBSS: Hanks’ Balanced Salt Solution
LPS: lipopolysaccharide
LY: Lucifer Yellow
OCLN: occludin
RFU: relative fluorescence units
TEER: trans-epithelial electrical resistance
TNF: tumour necrosis factor
TNSS: total nasal symptom score
